# SIRM–SIC appropriateness criteria for the use of Cardiac Computed Tomography. Part 1: Congenital heart diseases, primary prevention, risk assessment before surgery, suspected CAD in symptomatic patients, plaque and epicardial adipose tissue characterization, and functional assessment of stenosis

**DOI:** 10.1007/s11547-021-01378-0

**Published:** 2021-06-23

**Authors:** Antonio Esposito, Marco Francone, Daniele Andreini, Vitaliano Buffa, Filippo Cademartiri, Iacopo Carbone, Alberto Clemente, Andrea Igoren Guaricci, Marco Guglielmo, Ciro Indolfi, Ludovico La Grutta, Guido Ligabue, Carlo Liguori, Giuseppe Mercuro, Saima Mushtaq, Danilo Neglia, Anna Palmisano, Roberto Sciagrà, Sara Seitun, Davide Vignale, Gianluca Pontone, Nazario Carrabba

**Affiliations:** 1 Clinical and Experimental Radiology Unit, Experimental Imaging Center, IRCCS Ospedale San Raffaele, Via Olgettina 60, 20132 Milan, Italy; 2 Vita-Salute San Raffaele University, Milan, Italy; 3 Department of Biomedical Sciences, Humanitas University, Milan, Italy; 4 Humanitas Research Hospital IRCCS, Rozzano, Milan, Italy; 5 Centro Cardiologico Monzino IRCCS, Milan, Italy; 6 Department of Clinical Sciences and Community Health, University of Milan, Milan, Italy; 7 Department of Radiology, Azienda Ospedaliera San Camillo Forlanini, Rome, Italy; 8 SDN IRCCS, Naples, Italy; 9 Department of Radiological, Oncological and Pathological Sciences, “Sapienza” University of Rome, Rome, Italy; 10 Fondazione Toscana G. Monasterio, Massa and Pisa, Italy; 11 Cardiothoracic Department, University Cardiology Unit, Policlinic University Hospital, Bari, Italy; 12 Department of Medical and Surgical Sciences, Magna Grecia University, Catanzaro, Italy; 13 Department of Health Promotion, Mother and Child Care, Internal Medicine and Medical Specialties-ProMISE, University of Palermo, AOUP P. Giaccone, Palermo, Italy; 14 Department of Medical and Surgical Sciences, Modena and Reggio Emilia University, Modena, Italy; 15 Radiology Department, AOU of Modena, Modena, Italy; 16 Radiology Unit, Ospedale del Mare- A.S.LNa1-Centro, Naples, Italy; 17 Department of Medical Sciences and Public Health, University of Cagliari, Cagliari, Italy; 18 Cardiovascular Department, CNR (National Council of Research)/Tuscany Region ‘Gabriele Monasterio’ Foundation (FTGM), Pisa, Italy; 19 Nuclear Medicine Unit, Department of Experimental and Clinical Biomedical Sciences “Mario Serio”, University of Florence, Florence, Italy; 20 Radiology Department, Ospedale Policlinico San Martino, IRCCS Per L’Oncologia E Le Neuroscienze, Genoa, Italy; 21 Cardiothoracovascular Department, Azienda Ospedaliero Universitaria Careggi, Florence, Italy

**Keywords:** Coronary CT angiography, Chest pain, Congenital heart disease, Epicardial adipose tissue, Plaque, Stenosis

## Abstract

In the past 20 years, Cardiac Computed Tomography (CCT) has become a pivotal technique for the noninvasive diagnostic work-up of coronary and cardiac diseases. Continuous technical and methodological improvements, combined with fast growing scientific evidence, have progressively expanded the clinical role of CCT. Recent large multicenter randomized clinical trials documented the high prognostic value of CCT and its capability to increase the cost-effectiveness of the management of patients with suspected CAD. In the meantime, CCT, initially perceived as a simple non-invasive technique for studying coronary anatomy, has transformed into a multiparametric “one-stop-shop” approach able to investigate the heart in a comprehensive way, including functional, structural and pathophysiological biomarkers. In this complex and revolutionary scenario, it is urgently needed to provide an updated guide for the appropriate use of CCT in different clinical settings. This manuscript, endorsed by the Italian Society of Medical and Interventional Radiology (SIRM) and by the Italian Society of Cardiology (SIC), represents the first of two consensus documents collecting the expert opinion of Radiologists and Cardiologists about current appropriate use of CCT.

## Introduction

**Fig. 1:**
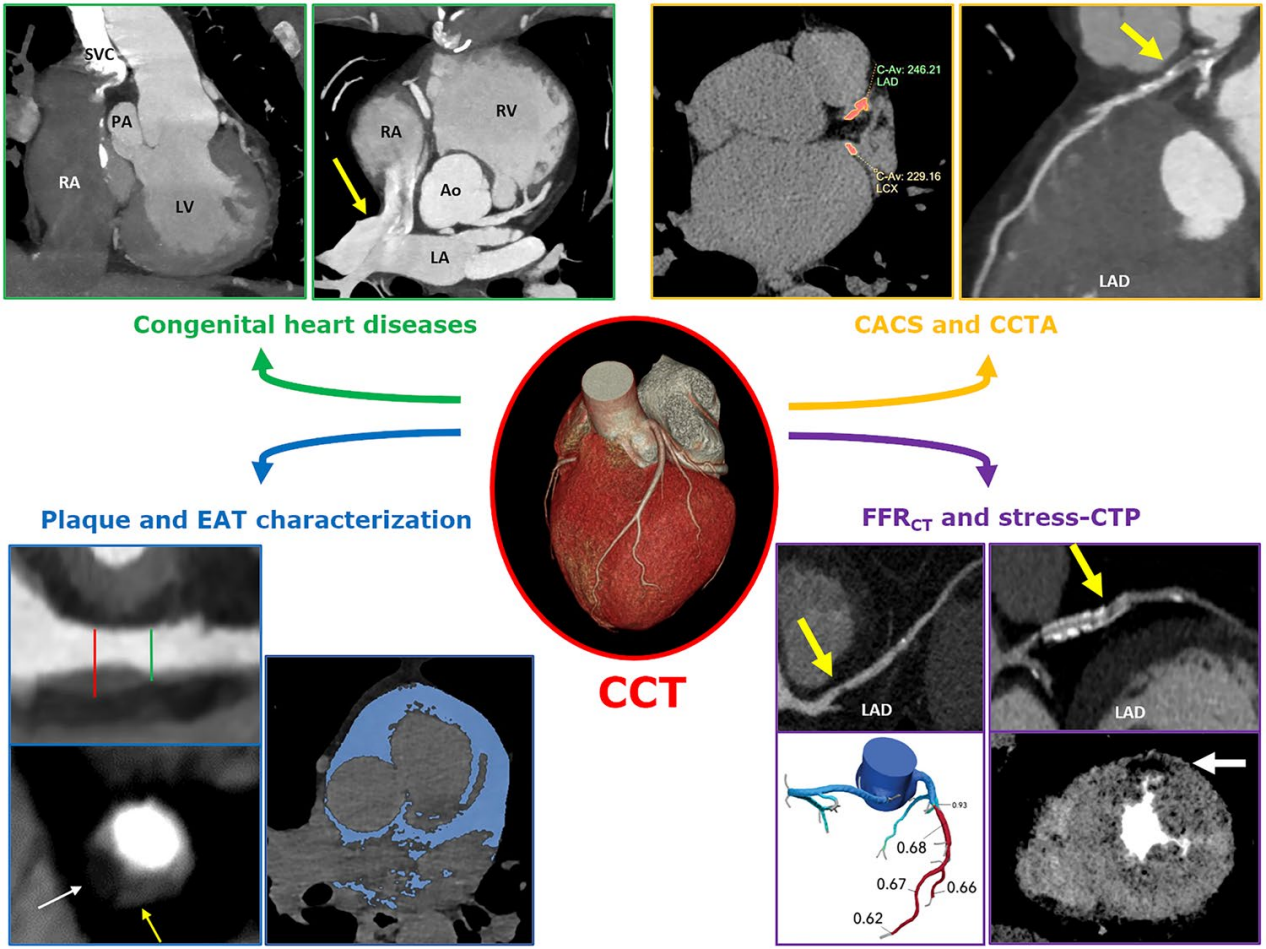
Graphical overview of the main applications of Cardiac Computed Tomography discussed in this part I appropriateness criteria guidelines from SIRM-SIC

Cardiac Computed Tomography (CCT) was historically adopted as a tool to rule-out coronary artery disease (CAD) due to the well-established very high negative predictive value. Recently, the results of multicenter randomized clinical trials have changed the perception of CCT in the clinical world, leading the scientific community to recognize CCT as the first line diagnostic test for most of the patients with suspected chronic coronary syndrome [1] and in some cases of acute chest pain presentation [2]. Moreover, due to technical improvements and scientific progress, CCT was promoted as a potential test to implement prevention strategies in some specific settings [3], and as an imaging tool able to characterize coronary plaques [4], myocardium [5] and epicardial fat [6]. Furthermore, different strategies were developed to integrate the outstanding anatomical data with functional information revealing the pathophysiological impact of a coronary stenosis [7] (Fig. 1).

In this complex and revolutionary scenario, in which guidelines help the translation of evidences into clinical practice [8], there is a clear need of updating the previously published documents on appropriateness for clinical/practical use of CCT [9–12].

This manuscript, endorsed by the Italian Society of Medical and Interventional Radiology (SIRM) and by the Italian Society of Cardiology (SIC), represents the first of two consensus documents collecting the expert opinion of Radiologists and Cardiologists about current appropriate use of CCT and integrates the guidelines for appropriate use of cardiovascular magnetic resonance (CMR) recently published by the same working group [13].

## Definition of appropriateness and applied methodology

The writing committee discussed the table of content and assigned referrals for each chapter.

Each referral conducted literature review and drafted the assigned section highlighting indications and rating them according to the following score:

A. Strong recommendation: there is evidence, general agreement, or both, that the test is useful (benefit >  >  > risk).
B. Moderate recommendation: there is conflicting evidence or opinion about the usefulness of the test; the weight of evidence/opinion, however, is strongly in favor of the test’s usefulness. (benefit >  > risk).
C. Weak recommendation: the test’s usefulness is less well established; there is a small net benefit (benefit ≥ risk)
D. No recommendation: there is evidence or general agreement that the risk/harm outweighs benefits (benefit = or < risk).
E. Expert opinion: there is insufficient evidence or evidence is unclear or conflicting, but this is what the working group recommends. Further research is recommended in this area.

Assigned scores were discussed in consensus by all authors and unanimously approved.

## Congenital heart diseases

In pre- and post-surgical complex congenital heart diseases (CHD), multimodality imaging is required for both the detailed evaluation of cardiovascular anatomy and for the functional characterization of cardiac chambers and flows. Catheterization is required for pulmonary vascular resistances calculation, whereas for most types of CHD and congenital coronary artery anomalies (CAAs) CCT is adopted as a complementary imaging modality [14] (Table 1).

**Table 1:** Congenital heart diseases

Clinical setting	Diagnostic step	Recommendation	Indication
**Coronary arteries anomalies**			
Isolated congenital coronary artery anomal﻿y	First diagnosis	A	Identification of coronary artery origin, course, angulation from the aortic root, ostial atresia, presence and length of intramural course, presence of arteriovenous fistula
	Follow-up	A	Worsening clinical status or new signs/symptoms Surveillance in patients with no or mild sequelae
**Conotruncal CHD**			
Tetralogy of Fallot (TOF)	First diagnosis	D	Not recommended
		A	Presence of associated major aortopulmonary collateral arteries (MAPCAs)
	Follow-up (initial repair)	A	Depiction of coronary arteries anatomy before pulmonary valve replacement
	Follow-up (postoperative)	A	In symptomatic patients or as surveillance in patients with no or mild sequelae especially when CMR is contraindicated
D-loop transposition of the great arteries	First diagnosis	D	Not recommended
	Follow-up (postoperative)	A	Evaluation of reimplanted coronary artery in asymptomatic and symptomatic patients Surveillance in patients with neoaortic root dilation In symptomatic patients or as surveillance in patients with no or mild sequelae especially when CMR is contraindicated
Truncus arteriosus	First diagnosis	A	Evaluation prior to surgery
	Follow-up (postoperative)	A	Surveillance in symptomatic patients or in asymptomatic patients with moderate or severe truncal stenosis or regurgitation
**Septal anomalies**			
Atrial septal defects (ASD) and partial anomalous pulmonary venous return (PAPVR)	First diagnosis	A	In patients with sinus venous defect and PAPVR for procedural planning
	Follow-up (postoperative)	C	In symptomatic patients or as surveillance in patients with no or mild sequelae
	Follow-up (unrepaired)	E	Surveillance in asymptomatic patients with moderate or severe ASD and PAPVR of > 1 pulmonary vein
Ventricular septal defects (VSD) and atrioventricular septal defects (AVSD)	First diagnosis	D	Not recommended
	Follow-up (postoperative)	D	Not recommended
**Mediastinal vessels anomalies**			
Aortic coarctation and aortic arch anomalies	First diagnosis	A	Evaluation prior to surgery
	Follow-up	A	Surveillance in patients with mild aortic coarctation Surveillance in asymptomatic patients after surgery
Total anomalous pulmonary venous return	First diagnosis	A	Evaluation and preprocedural planning
	Follow-up (postoperative)	B	Surveillance in patients with no or mild sequelae
Vascular rings and pulmonary artery slings	First diagnosis	A	Vascular and tracheobronchial anatomy depiction and preprocedural planning
	Follow-up (postoperative)	B	Surveillance in patients with no or mild sequelae
**Single-ventricle heart disease**			
Functional single ventricle	First diagnosis	A	Evaluation prior to stage 1 palliation
After stage 1 palliation (e.g., systemic-to-pulmonary artery shunt, patent ductus arteriosus stent)	Surgical planning and follow-up	A	Evaluation prior to stage 2 and stage 3 palliation Surveillance in patients with no or mild sequelae

Echocardiography is the initial imaging tool for morpho-functional evaluation; however, a frequently limited acoustic window hampers the assessment of the mediastinal vessels, extra-cardiac surgical conduits, and intra-cardiac complex anatomy, particularly in adults with grown-up congenital heart diseases (GUCH).

The use of CCT has been described in patients of all ages and with CHD of all levels of complexity, especially when echocardiography is not exhaustive. CCT is generally recommended in complex conditions that require investigation of coronary vessels or complex vascular and thoracic anatomy [15, 16]. CCT provides high anatomical detail about pulmonary vessels, when compared to surgical findings [17], and about aorto-pulmonary collaterals prior to surgery in patients with pulmonary atresia, septal defects, and major aorto-pulmonary collateral arteries.

In patients with suspected vascular rings and slings or tracheobronchial narrowing for complete cartilaginous rings, CCT is the method of choice for the pre-surgical evaluation of tracheobronchial tree and pulmonary parenchyma [16]. Congenital coronary anomalies are relatively common in patients with Tetralogy of Fallot, and the definition of origin and course prior to surgery is needful, particularly in patients with an anomalous coronary that crosses the right ventricle outflow tract [18].

CMR remains the method of choice in the follow-up of complex CHD due to the absence of ionizing radiation and for its capability to quantify vessel flows and ventricular function and to identify myocardial fibrosis. However, CMR is time consuming and image quality may be reduced in patients with metallic devices. CCT provides better visualization of stents, conduits, and metallic objects and is safe in patients with implanted pacemakers and defibrillators [19]. Moreover, CCT can measure bi-ventricular volumes and function with very high accuracy when scanners with adequate temporal resolution are adopted. Therefore, CCT plays an important role in the follow-up of adult patients with GUCHs who cannot undergo CMR [20].

Finally, CCT may provide useful morphological information to avoid external coronary artery compression related to device release in transcatheter pulmonary valve replacement [21] and to identify sub-sternal course of coronary arteries before repeated sternotomy [22].

The main limitation of CCT is ionizing radiation exposure; however, low-dose acquisition protocols can be adopted [23].

## Primary prevention in asymptomatic patients

### Coronary artery calcium scoring

Coronary Artery Calcium Score (CACS), reported as Agatston score [24], measures the amount of calcium in the coronary arteries and is a surrogate marker for atherosclerotic burden. CACS predicts the risk of events in asymptomatic individuals independently of the presence of obstructive CAD [25]. A proportional relationship between stratified CACS (0, 1–99, 100–399 and ≥ 400), total atherosclerotic plaque burden [26], and outcome has been found [27] (Table 2).

**Table 2:** Primary prevention in asymptomatic patients—Coronary Artery Calcium Score (CACS) and coronary CT angiography (CCTA)

Clinical setting	Diagnostic step	Recommendation	Indications
CACS in patients with low risk of CAD	First diagnosis	B	In 40-to-75 years old patients with strong family history of premature CAD
CACS in patients with intermediate risk of CAD	First diagnosis	A	In 40-to-75 years old patients If CACS = 0, no statin or aspirin required unless persistent smoker or strong family history of CAD
CACS in patients with high risk of CAD	First diagnosis	D	Not recommended
CACS in patients with diabetes	First diagnosis	B	In > 40 years old patients
Repeated CACS	Follow-up	B	At 5 years in patients with CACS = 0 At 3-to-5 years in patients with CACS > 0 or diabetes
CCTA after CACS for CAD screening	First diagnosis	B	In patients with CACS in the range 101–400
CCTA for CAD screening	First diagnosis	D	Extensive screening is not recommended
		B	Screening in high-risk populations (e.g., patients with diabetes, patients with familial hypercholesterolaemia) Screening in specific populations (e.g., pre-participation screening of athletes > 35 years old, specific jobs such as in aviation)
	Follow-up	A	Follow-up of heart transplantation

Recent studies [28, 29] have shown the additional value of CACS beyond traditional risk factors, supporting the integration of CACS into cardiovascular risk assessment. The 2016 European Society of Cardiology (ESC) guidelines for cardiovascular disease prevention gave a class II recommendation for CACS in intermediate-risk patients [30]. The 2019 ESC guidelines for chronic coronary syndromes gave CACS a IIb recommendation for screening asymptomatic patients [1], with particular value as a risk modifier in patients with intermediate (5–15%) pre-test probability (PTP) [1].

CACS may have a role also in individuals aged 45-to-75 years with low cardiovascular risk but with strong family history of premature CAD and in diabetics patients aged > 40 years or at intermediate-risk of early CAD [31, 32].

The absence of CACS carries a favorable 5-year and 15-year prognosis for patients with and without diabetes, respectively [33].

Finally, CACS is considered useful for guiding preventive medical therapy [34], avoiding misclassifications and under- or over-treatment [35]. As a result, according to AHA and ACC guidelines for the management of blood cholesterol, CACS assessment is considered crucial to decide if starting statin therapy [36].

### Coronary CT angiography

According to 2019 ESC guidelines on chronic coronary syndromes, coronary CT Angiography (CCTA) is not recommended for extensive screening of asymptomatic individuals [1]. However, CCTA has an incremental prognostic value over the Framingham risk score for prediction of mortality and non-fatal myocardial infarction in asymptomatic individuals with CACS from 101 to 400 [37]. Moreover, it may be reasonable to consider CCTA in selected subgroups of asymptomatic patients at high risk of coronary events, such as diabetic patients. In this setting, CCTA identifies patients at increased risk of cardiac events with incremental value over clinical risk assessment and CACS [38]. However, RCT and meta-analysis showed that CCTA does not significantly reduce major adverse cardiovascular events (MACEs) [39] or the rate of non-fatal myocardial infarction and hospitalization for heart failure [40], even if it significantly reduces the rate of any cardiac event [40]. Furthermore, in high risk patients, CCTA promotes a more aggressive modification of risk factors and medical or revascularization therapy [41].

This is reflected in 2019 ESC guidelines that suggest that asymptomatic diabetic subjects with CACS > 400 may be referred for functional imaging or CCTA [32]. However, inherent limitations of CCTA in patients with heavily calcified coronary arteries [42] and local technological level and operator expertise should be taken into account.

Finally, some evidence suggests that CCTA could enhance screening in asymptomatic individuals in specific sporting (i.e., pre-participation screening of athletes aged > 35 years or in young athletes for the exclusion of significant CAD or coronary anomalies) [43, 44] or working (i.e., aviation personnel) [45] settings (Table 2).

## Risk assessment before major surgery

### Non-cardiac major surgery

Non-cardiac surgery is associated with an incidence of complications from 7 to 11%, with a mortality rate of 0.8% to 1.5%, largely driven (42% of cases) by cardiac complications. Based on the rate of cardiovascular events (death or myocardial infarction within 30 days from surgery), surgical procedures are classified at low, intermediate, or high risk (< 1%, 1–5%, and > 5%, respectively). The current guidelines recommend coronary functional testing for patients with an unknown or impaired functional status undergoing intermediate-to-high risk non-cardiac planned surgery [46, 47]. Nevertheless, the capability to predict MACEs within 30 days from non-cardiac surgery remains limited [48].

In a recently published meta-analysis, CCTA was found to safely predict freedom from perioperative MACEs in a cohort of patients at high risk according to clinical indices [49]. The severity and extent of CAD improved risk stratification, and multivessel disease was associated with the highest risk (OR 8.9). Similarly, increasing CACS was associated with higher risk of perioperative MACEs (CACS ≥ 100, OR 5.1; CACS ≥ 1000, OR 10.4) [49]. Given its well-know very high NPV, CCTA is recommended in patients with low-to-intermediate risk of CAD undergoing high risk surgery, particularly if unable to take functional stress testing or with inconclusive findings. Nevertheless, further trials are needed to better identify the subclasses of patients getting the higher value from a functional or anatomical approach in this specific setting (Table 3).

**Table 3:** CCTA-based risk assessment before major non-cardiac and cardiac surgery

Major surgery			
Clinical setting	Diagnostic step	Recommendation	Indications
Low-to-intermediate surgical risk	First diagnosis	D	Not recommended
High surgical risk	First diagnosis	B	In low risk of CAD
		A	Intermediate risk of CAD
		E	In high risk of CAD
Cardiac valvular surgery	First diagnosis	A	Patients with suspected ischemia, systolic disfunction, male > 40 years, post-menopausal women, patients with ≥ 1 risk factors

### Cardiac surgery

CAD needs to be screened in patients scheduled for cardiac surgery for pre-operative risk assessment. In particular, a thorough cardiological evaluation is indicated in patients with severe valve disease with history of CAD, suspected ischemia, systolic dysfunction, male with age > 40 years, post-menopausal women, and patients with one or more risk factors. Several studies indicated that CCTA can reliably replace invasive coronary angiography (ICA) as a screening tool before valve interventions [50–52], especially in patients at low-to-intermediate risk of CAD, and in patients at high risk of ICA-related complications (i.e., aortic dissection, valve vegetations, prosthetic thrombosis) (Table 3).

## Suspected CAD in symptomatic patients

In the recent past, stable symptomatic patients with chest pain were non-invasively assessed using different functional tests, including mainly treadmill testing, stress echocardiography and single photon emission computed tomography (SPECT). Stress perfusion cardiac magnetic resonance (stress-CMR) and positron emission tomography (PET) were less used due to availability and costs concerns, albeit showing a higher diagnostic accuracy. Unfortunately, despite routine use of these tests, only one-third of the patients with a positive functional test turns out to be affected by obstructive CAD at ICA [53], revealing a high rate of false-positive or undetermined results of these non-invasive functional tests (Table 4).

**Table 4:** CCTA in symptomatic patients with suspected CAD

Clinical setting	Diagnostic step	Recommendation	Indication
Patients with conditions that likely hamper image quality	First diagnosis	C	The imaging modality with higher cost-effectiveness should be identified case by case for difficult patients because conditions that likely hamper image quality in CT (e.g., high-grade obesity, limited compliance) may also hamper feasibility of different functional imaging modalities Extensive coronary calcifications or highly irregular heartbeat should suggest considering other imaging modalities
Patients with low-to-intermediate pre-test likelihood of CAD	First diagnosis	A	As first line test
Patients with high pre-test likelihood of CAD	First diagnosis	B	As first line test
Patients with very high pre-test likelihood of CAD	First diagnosis	D	Not recommended
Patients with low pre-test likelihood of CAD	First diagnosis	A	After positive appropriate functional stress test
		C	After negative appropriate functional stress test
Patients with high pre-test likelihood of CAD	First diagnosis	C	After positive appropriate functional stress test
		A	After negative appropriate functional stress test
Regardless of pre-test likelihood of CAD	First diagnosis	A	After equivocal or uninterpretable appropriate functional stress test After two or more appropriate functional stress test with opposite results
Patients with suspected vasospastic angina	First diagnosis	A	To determine the extent of underlying CAD

In the recent years, CCTA was found to detect with high accuracy non-obstructive CAD defined by ICA, and to reduce unnecessary ICAs when compared to functional testing [54].

The prognostic value of CCTA in stable symptomatic patients is no longer debated since the publication of the results of the PROMISE [54] and the SCOT-HEART [55] trials. CCTA is highly effective as a guide to enhance risk factors modification and preventive therapy adoption [56]. CCTA was found to reduce the rate of events when performed in addition to routine test [55, 56], to provide outcome information comparable to functional imaging [57], and, when associated with non-invasive fractional flow reserve (FFR_CT_), it is comparable to ICA with invasive FFR in targeted revascularization [58].

In line with these evidence, the latest update of the National Institute for Health and Care Excellence (NICE) clinical guidelines [59] and the 2019 ESC guidelines for the diagnosis and management of chronic coronary syndromes [1] recommended CCTA as the initial test to rule-out CAD in patients in which obstructive CAD cannot be excluded by clinical assessment alone (Class I). CCTA should be also considered as an alternative to ICA for non-diagnostic or indeterminate results of other noninvasive tests (Class IIa).

For stenosis estimated to be in the range 50–90% at CCTA, functional significance should be considered uncertain [60] [61], being inducible ischemia found in approximately 50% of patients with obstructive CAD at CCTA (≥ 50%); hence, myocardial ischemia test is recommended as in the case of non-diagnostic CCTA (Class I) [1].

In past studies adopting old technology, it was found that the accuracy of CCTA was influenced by the pre-test probability (PTP) of CAD [62], being particularly high for patients with low-to-intermediate PTP of CAD [63, 64] driven by the very high NPV of CCTA [65–68]. Recent technological advancement, with improvement of spatial and temporal resolution, has led to a significant improvement also of the PPV and of the specificity [69–72]. These findings, associated with the tendency of clinical risk scores to overestimate the pre-test probability of obstructive CAD [1, 73, 74], led to consider CCTA irrespective of PTP, with the exception of patients with very high PTP (> 90%) in whom ICA is indicated, and for patients with very low clinical likelihood (≤ 5%), in whom no further test is indicated (ESC 2019).

However, CCTA is to be avoided in the presence of conditions which cannot ensure good image quality related to local availability and expertise, scanner technology, and patient characteristics, including extensive coronary calcification, irregular heart rate, severe obesity, and inability to breath-hold (Class III) [1].

## Coronary atherosclerotic plaque and epicardial adipose tissue characterization

CCTA has the unique capability to non-invasively quantify coronary atherosclerosis and to characterize plaque morphology and composition with high accuracy compared to histology and intravascular ultrasound (IVUS) [75]. This is important for risk stratification and has the potential advantage to guide preventive therapy [56] and to assess treatment efficacy [76] (Table 5).

**Table 5:** Coronary Atherosclerotic Plaque and Epicardial Adipose Tissue (EAT) characterization

Clinical setting	Diagnostic step	Recommendation	Indication
Plaque imaging	First diagnosis	B	Classification of plaques as soft, calcified, or mixed Identification and description of high-risk plaque features
	Follow-up	C	Classification of plaques as soft, calcified, or mixed Identification and description of high-risk plaque features
Epicardial adipose tissue (EAT)	First diagnosis	E	Measuring of EAT volume and attenuation is not currently clinically indicated. Interesting tool needing further research
	Follow-up	E	Measuring of EAT volume and attenuation is not currently clinically indicated. Interesting tool needing further research

Patients with obstructive CAD have worse outcomes compared to patients with nonobstructive or absent CAD [77]. However, most of acute coronary syndromes arise from nonobstructive plaques with vulnerable features [78]. CCTA can identify high-risk plaques (HRP) by evaluating several features such as the napkin ring sign (thin overlying fibrous cap), positive vessel remodeling (ratio between lesion diameter and reference diameter > 1.1), low attenuation (< 30 HU), and spotty calcifications (focal calcification within the coronary artery wall < 3 mm in maximum diameter). Recent trials [55, 79–81] highlighted that evaluation of non-obstructive HRPs has incremental prognostic value in predicting coronary events [82] beyond cardiovascular risk factors and obstructive CAD presence [79, 81].

Other plaque characteristics, such as extent and location (proximal vs distal), have been associated with clinical outcome [81, 83], and percent atheroma volume and stenosis diameter have been found as independent predictors for the development of obstructive CAD [84]. Furthermore, it has been shown that, after an acute coronary syndrome, predictors of future events are large plaque burden and lipid-rich lesions, which can be assessed by CCTA. For this purpose, comprehensive CT-based scores (such as the CT-Leaman score) have been created and proved to be associated with future cardiovascular events [85].

These data support the reporting of HRP features presence (if more than 2 HRP features are evident) even for non-obstructive lesions, as suggested by CAD-RADS guidelines [86].

Some technical limitations may impact on CCTA-based plaque characterization, most importantly spatial resolution and other factors such as a certain degree of density overlap in lipid-rich and fibrous-rich non-calcified plaques. Dual-energy CT may overcome these limitations thanks to tissue decomposition algorithms. However, this approach is still limited to research and initial results need to be validated [87].

Pericoronary adipose tissue is emerging as an imaging biomarker to identify plaque instability. Increased epicardial adipose tissue (EAT) attenuation was detected around inflamed plaques [88]. Moreover, EAT can modulate coronary artery function through paracrine and vasocrine pathways by producing cardioprotective adipokines in physiological conditions or a pro-atherogenic secretome in case of dysfunction [89]. EAT volume and its attenuation properties can be quantified by CCTA [88, 90]. In a recent study [89], an alteration of EAT attenuation was found to be associate with non-calcified and vulnerable plaques in early CAD, while in advanced CAD it was found that EAT exhibits pro-calcifying properties. The role of EAT volume and attenuation has been investigated in sparse studies and its association with CAD and outcome remains uncertain. This is mainly due to the paucity of available data, heterogeneous methodology, small sample size, and different clinical setting.

## CT-derived fractional flow reserve (FFR_CT_) and stress computed tomography perfusion (stress-CTP)

FFR_CT_ and stress-CTP allow to integrate information about the hemodynamic significance of coronary lesions to angiographic evaluation of CAD, thus potentially avoiding additional examinations and costs. In fact, data coming from iFFR show that only 35% of anatomically obstructive lesions have positive iFFR reflecting hemodynamic significance of a stenosis. Thus, iFFR is a key parameter to guide revascularization, improving the outcome and reducing health care costs [91, 92] (Table 6).

**Table 6:** Recommendations for CT-derived Fractional Flow Reserve (FFR_CT_) and stress-CT perfusion (stress-CTP)

Clinical setting	Diagnostic step	Recommendation	Indications
FFR_CT_ for evaluation of CAD	First diagnosis	E	Very promising in: CAD with suspected functional significance at CCTA CAD with uncertain functional significance at CCTA (especially intermediate or calcified lesions) Evaluation of hemodynamic significance of triple vessel disease However, current limited availability of validated analysis platforms hampers widespread clinical application
Stress-CTP for evaluation of CAD	First diagnosis	E	Very promising in: CAD with suspected functional significance at CCTA CAD with uncertain functional significance at CCTA Evaluation of hemodynamic significance of triple vessel disease However, current lack of methodological standardization, limited validation data, technological requirements, and dose concerns hamper widespread clinical application

Computational fluid dynamics allows to noninvasively estimate the FFR from CCTA [93], which has been extensively validated against iFFR in three multicentre studies [94–96]. Also an improvement in specificity and diagnostic accuracy in comparison with CCTA alone has been reported [94–96]. The high diagnostic accuracy of FFR_CT_ is maintained also in patients with intermediate stenosis and in the presence of calcified plaques [97] or 3-vessel CAD [98].

FFR_CT_ modifies treatment in two-thirds of subject compared to CCTA alone [91], safely reducing unnecessary ICA [92], and predicts the outcome at 1- and 5-years [99].

Despite the advantages, FFR_CT_ analysis is currently time consuming (2–6 h) due to software constraints and offsite analysis [100] and currently there is only one commercially available algorithm (Heart-Flow Inc., Redwood, CA). Furthermore, performance of FFR_CT_ is strictly related to image quality. Imaging artifacts caused by low contrast, cardiac and respiratory motion, blooming due to severe calcification, and image noise due to low radiation exposure or high body mass index hamper FFR_CT_ performance [100].

Also, stress-CTP is capable of detecting functionally relevant stenosis, improving the diagnostic performances of CCTA, with similar performance in comparison with CCTA combined with FFR_CT_ [101].

Stress-CTP depicts perfusion defects as a hypo-attenuating myocardial region using either a static protocol (single scan acquired both at rest and during stress at the peak of iodine concentration in the coronaries) or a dynamic protocol in which several datasets are acquired during first pass perfusion. Dynamic stress-CTP has the advantage of providing quantitative evaluation of perfusion by estimating the myocardial blood flow [102].

Regardless of the acquisition protocol, stress-CTP requires the administration of pharmaceutical stressors and notably increases both radiation exposure and iodinated contrast agent dose.

Stress-CTP shows similar performance with respect to stress-CMR (AUC 0.91 vs 0.95 at per-patient and 0.88 vs 0.93 at per-vessel analysis) and slightly better performance than single photon emission computed tomography (AUC 0.91 vs. 0.87) [103] and has reasonably high sensitivity and specificity (88% and 80%, respectively) in detecting flow-limiting coronary stenosis using iFFR as reference standard [104]. Importantly, the use of stress-CTP in patients at intermediate-to-high-risk of CAD was shown to improve the diagnostic performance of CCTA from 83 to 93% in a per vessel analysis [105].

However, these data need to be confirmed on larger populations. Furthermore, differently from FFR_CT_, data on clinical utility and outcome have not been reported [106].
